# Dynamic and Static Switching in ITO/SnOx/ITO and Its Synaptic Application

**DOI:** 10.3390/ijms23179995

**Published:** 2022-09-02

**Authors:** Jongmin Park, Hyunwoong Park, Daewon Chung, Sungjun Kim

**Affiliations:** Division of Electronics and Electrical Engineering, Dongguk University, Seoul 04620, Korea

**Keywords:** RRAM, dynamic, static, conduction mechanism, synaptic application

## Abstract

The attempts to devise networks that resemble human minds are steadily progressing through the development and diversification of neural networks (NN), such as artificial NN (ANN), convolution NN (CNN), and recurrent NN (RNN). Meanwhile, memory devices applied on the networks are also being studied together, and RRAM is the one of the most promising candidates. The fabricated ITO/SnOX/TaN device showed two forms of current–voltage (I-V) curves, classified as dynamic and static. It was triggered from the forming process, and the difference between the two curves resulted from the data retention measured at room temperature for 10^3^ s. The dynamic curve shows a time-dependent change in the data, and the cause of the data preservation period was considered through X-ray photoelectron spectroscopy (XPS) and linear fitting in conduction mechanisms. To confirm whether the memory performance of the device may be implemented on the synapse, the change in the plasticity was confirmed using a rectangular-shaped pulse. Paired-pulse facilitation (PPF) was implemented, and the change from short-term potentiation (STP) to long-term potentiation (LTP) was achieved.

## 1. Introduction

Even though device contraction has reached its limit, interest in integration technologies such as vertical stacking and packaging are increasing day by day to meet consumers’ increasing needs. Traditional devices, such as DRAM and Flash memory, show that stable and fast operation are also being vertically stacked to meet the needs of the markets [1,2,3,4,5]. However, since the scaling-down issue of the capacitor and the complex structure of the transistor prevent the increase in integration density, the next-generation memory needed to break the limit is being studied. Among the next-generation memories, resistive random-access memory (RRAM) has a simple, metal–insulator–metal (MIM) structure so it can be easily applied to existing fabrication processes and has excellent integration advantages. It also shows fast switching speed encouraged by the removal of the capacitor and has a low operating voltage that makes it suitable for neuromorphic array configuration that requires low power consumption [6,7,8,9,10].

Research on the practical use of tin oxide (SnO_X_) has been conducted by many groups in applications in gas sensors and n-type thin-film transistors. In particular, it is used as an electrode of a solar cell in recognition of its potential as a transparent electrode due to its high transparency. The wide bandgap of ~3.6 eV of SnO_X_ makes its application useful in the field of RRAM, and related research is being conducted [11,12]. Unlike conventional SnO_X_, which demands an annealing process to achieve resistive switching (RS) characteristics, the as-deposited SnO_X_ used in this paper showed RS characteristics. As-deposited SnO_X_ by direct current (DC) reactive magnetron sputtering has already been verified the RS in a Ag/SnO_X_/ITO device by Da chen and Shi-Hua Huang, and it showed a low resistance of less than 3 kΩ and a resistance window of less than ~10 [13,14]. In addition, Jidong Jin et al. reported that the DC-sputtered SnO_X_ showed poor RS on the stack of an Al/SnO_X_/Pt device [15]. Our DC-sputtered SnO_X_ was fabricated at room temperature and showed normal RS behavior.

In recent years, artificial neural networks with various types of concepts and applications have been studied. Due to this influence, the memory device has started to be divided into two branches of static device and dynamic device. Unlike traditional static devices that produce only the data of ‘0’ and ‘1’ in accordance with the electrical pulses, the dynamic devices are influenced by spatiotemporal signals that the conductance is time-dependent on decay characteristics [16,17,18,19]. The ITO/SnO_X_/TaN device showed two distinct forms of I–V curves and different temporal dependence of retention triggered by the forming process. Different conduction mechanisms were applied on each curve through linear fitting, and we considered the switching mechanism of the dynamic curve. A resistance of more than about 1 kΩ and a resistance window of about 100 times were extracted from the read voltage. We confirmed that simple synaptic functions, such as paired-pulse facilitation (PPF), could be implemented in the dynamic curve, and the transition from short-term plasticity (STP) to long-term plasticity (LTP) was based on the excessive electrical stimulation [20,21,22,23,24].

## 2. Results and Discussions

**Figure 1:**
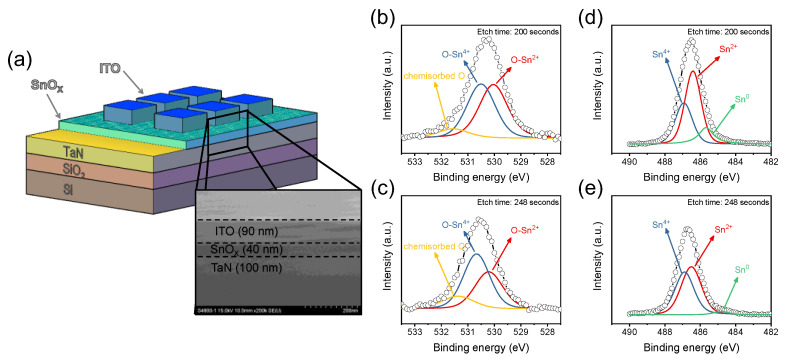
(**a**) The schematic image of ITO/SnO_X_/TaN device and FE-SEM image includes the thickness information of each layer. The O 1s core-level spectra of (**b**) ITO/SnO_X_ interface and (**c**) SnO_X_/TaN interface. The Sn 3d_5/2_ core level spectra of (**d**) ITO/SnO_X_ interface and (**e**) SnO_X_/TaN interface. The XPS analysis was conducted through the Shirley background and GL(10).

The schematic and FE-SEM image of ITO/SnO_X_/TaN device is shown in Figure 1a. The bottom electrode (BE) is in contact with the probe tip that maintains the ground bias, while DC and AC voltages are put into the top electrode (TE). The FE-SEM image shows the information of the thickness of each layer; a SnO_X_ of about 40 nm and ITO of about 90–100 nm were well-deposited by sputtering. To find out the atomic bonding of thin films at a specific point, XPS was investigated according to the depth mode. The depth mode was conducted by etching from TE to BE, and atomic distribution was confirmed that etch times of 200 s and 248 s, respectively, correspond to the interface of SnO_X_ between TE and BE. The Sn 3d_5/2_ in Figure 1b,c were fitted to ascribe the three components of Sn^4+^ at 486.8 eV, Sn^2+^ at 486.1 eV and Sn^0^ at 485.0 eV. In addition, the O 1s core-level XPS spectra in Figure 1d,e include the information about oxygen atoms binding with Sn. Like Sn 3d_5/2_, it was deconvoluted into three components of O-Sn^4+^ at 530.6 eV, O-Sn^2+^ at 530.0 eV and chemisorbed oxygen at 532.0 eV [25,26]. The common finding from Figure 1b–e is that the peak intensity of O-Sn^4+^ increases further at an etch time of 248 s, suggesting that two types of SnO_X_ existed in the thin film. The area ratio between Sn^2+^ and Sn^4+^ in Table 1 also indicates the increase in O-Sn^4+^ bonds. It can be divided into tin monoxide (SnO) derived from O-Sn^2+^ and tin dioxide (SnO_2_) derived from O-Sn^4+^. Khan et al. reported that increasing the SnO phase in SnO_2_ thin films reduces oxygen vacancies and decreases carrier concentration, resulting in an increase in resistivity [27]. Therefore, Sn^4+^ contributes to the conductivity of SnO_X_ and it influences the flow of electrons.

**Figure 2:**
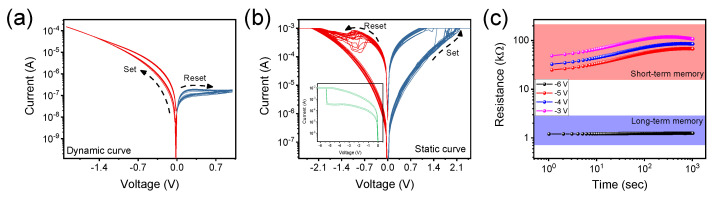
(**a**) The dynamic curves of ITO/SnO_X_/TaN device, which switched before the forming process. (**b**) The static curves of ITO/SnO_X_/TaN device, which switched after the forming process. The inset image shows the forming process. (**c**) The retention in the room temperature for 10^3^ s. The application regions were divided into short-term and long-term.

The function of long-term memory (LTM) for outputting data of ‘0’ and ‘1’ and the function of short-term memory (STM) for displaying decay characteristics through spatiotemporal pulse are divided by the artificial occurrence of the forming process. Figure 2a shows the dynamic I-V curves of a device reacted by a negative voltage, and it proceeded in the clockwise direction. Meanwhile, the forming process of the ITO/SnO_X_/TaN device is shown in the inset of Figure 2b, and Figure 2b shows that static I-V curves operated in the counterclockwise direction. Unlike static curves in which resistance was converted to the low state by negative voltage, the resistance window of dynamic curves increased as the magnitude of the negative voltage increases. This can be seen from the retention in Figure 2c, which was measured at room temperature for 1000 s. A read voltage of 0.5 V was used after giving the double linear sweep of expressed negative voltage, and the states were decayed over time in the STM region and maintained in the LTM region.

**Figure 3:**
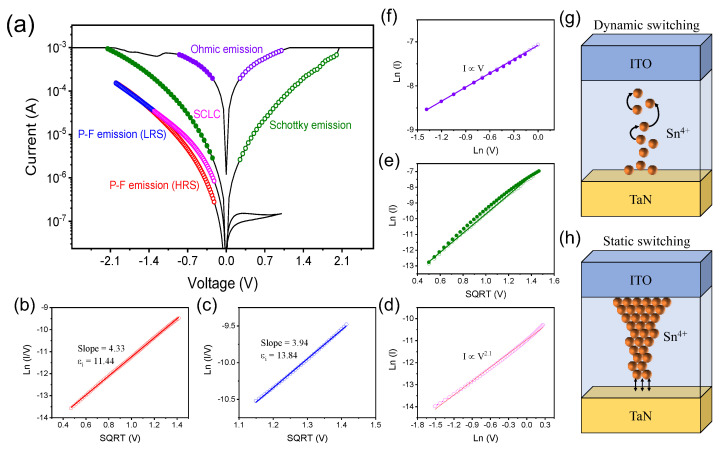
(**a**) Expressed conduction mechanism according to the linear fitting on the dynamic and static curves. The Pool–Frenkel emission in the dynamic curve of the (**b**) HRS and (**c**) LRS. (**d**) The LRS in the dynamic curve dominated by space charge-controlled current followed the Mott–Gurney law (I~V^2^). The linear fitting of (**e**) Schottky emission and (**f**) Ohmic conduction (I~V^2^) in the static curve. (**g**) Filamentary model of dynamic switching with P-F emission. (**h**) Filamentary model of static switching with Schottky emission.

The change in the I-V curves indicates that the direction of applied voltage makes a significant contribution on the operation of the device and conduction mechanism [28,29]. To figure out the conduction mechanism on each curve, we conducted linear fitting and confirmed that there was a difference between the dynamic and static curves as shown in Figure 3a. In Figure 3b, the linear plot of ln(I/V) versus V^1/2^ for Pool–Frenkel (P-F) emission in a high-resistance state (HRS) was well-fitted so that the electrons captured by Sn^4+^ were thermally excited into the conduction band. The expression of P-F emission is below [30]:

(1) $$J=q\mu N_{C}Eexp\left[\frac{-q\left(\phi_{T}-\sqrt{qE/\pi \varepsilon_{i}\varepsilon_{0}}\right)}{kT}\right]$$

where μ is the electron drift mobility, $N_{C}$ is the density of states in conduction band, $-q\phi_{T}$ is the trap energy, E is the electric field across the dielectric, k is the Boltzmann’s constant, T is the absolute temperature, $\varepsilon_{i}$ is the dielectric constant, and $\varepsilon_{0}$ is the permittivity in the vacuum. Based on the equation and linear fitting line, the $\varepsilon_{i}$ was calculated as 11.4, which is consistent with the values of 9–14 declared in the paper [31,32,33]. When the voltage was swept back in the low-resistance state (LRS), the most linearity could also be achieved in the plot of ln(I) versus V as shown in Figure 3c and showed the $\varepsilon_{i}$ of 13.8. This state was maintained until the voltage reaches from −2.0 V to −1.3 V, before it conformed to the Mott–Gurney law (I~V^2^) at the low electric field and governed by space charge-controlled current [34,35]. This is due to the filled trap sites in SnO_X_; the injected carriers from the anode are much more than thermally excited electrons. After the device goes through the forming process, the results on linear fitting are completely transformed such that HRS and LRS are converted into Schottky emission and Ohmic conduction, respectively. The immense forming voltage induces the decomposition of O-Sn^4+^ bonding and generates the large amount of interstitial defect states below the conduction band. The interstitial defect states lower the Schottky barrier, located between TaN and SnO_X_, and transform the conduction mechanism from P-F emission to Schottky emission [36]. The process of forming produces an excessive number of defect sites and has a profound effect on the performance of memory. The filamentary models according to the direction of the electric field and each conductive mechanism are shaped in Figure 3g,h. In dynamic switching, Sn^4+^ ions are migrated along the electric field toward the ITO electrode. However, after the forming voltage induces a highly conductive filament, it converts into static switching and performs a Schottky emission.

**Figure 4:**
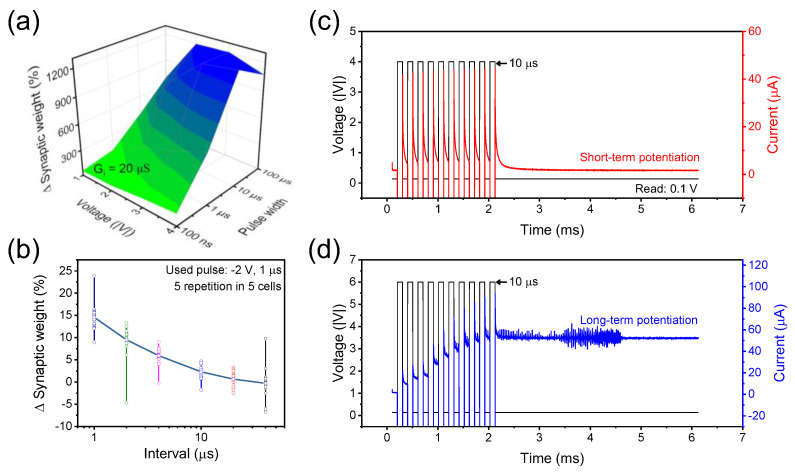
(**a**) The investigation of conductance value affected by the strength of the pulse. The bluer the color, the weight is more strengthened. And the greener the color, the weight is less strengthened. (**b**) The synaptic function called PPF, which was implemented through the paired pulses (−2 V, 1 μs). The potentiation of (**c**) short term and (**d**) long term. Pulses with different intensities can be used in different applications.

Before trying to imitate the synaptic functions in accordance with the above dynamic and static curves, we investigated the change in conductance with the rectangular-shaped electrical pulse; the strength of pulse was adjusted through the voltage size and the width. As shown in Figure 4a, the size was increased from 1 V to 4 V and the width was increased from 100 ns to 10 μs. The read process was performed at 0.1 V and the calculated conductance started from 20 μS increased by 12 times according to the intensity of the pulse. The PPF in Figure 4b was conducted in the dynamic curve and confirmed that the decrease in plasticity facilitation became severe when the gap between paired pulses (−2 V, 1 μs) widened. It was conducted in five different cells five times and showed that the conductance hardly increases after the gap exceeds 40 μs. The STM function was implemented in the dynamic curve, and the function conversion from STM to LTM could be confirmed through Figure 4c,d. The same 10 pulses but different voltage was used. No conversion occurred at the voltage of −4 V, but with the use of −6 V, the current gradually raised and then achieved. In conclusion, it is in common with the DC retention in Figure 2c and ITO/SnO_X_/TaN device is suitable as a device for composing the neural networks.

## 3. Materials and Methods

The ITO/SnO_X_/TaN RRAM device was prepared in the patterning size of 100 × 100 μm^2^ on the Si substrate with the thermally grown SiO_2_ of 300 nm, and it was pre-sputtered (GMEK Korea Inc., Anyang, Korea) TaN electrodes were located. The SnO_X_ and ITO were deposited through DC reactive sputtering, and ITO was lifted off after the patterning process. We used the Sn metal target (99.99%, 3 inch), while adding 10 sccm of Ar gas and 15 sccm of O_2_ gas for reactive reaction. The DC power of 50 W was delivered to the metal target and after pre-sputtering for about 10 min, the thickness of 40 nm was deposited at the rate of 1.45 Å/s. The deposition of ITO (90:10 wt%, 99.99%) was also conducted through a similar process with SnOX; the difference is that 90 W of DC power was applied and a reduced amount of O_2_ gas of 1 sccm was used with the Ar gas of 10 sccm. The ITO showed a deposition rate of 1.73 Å/s, and a thickness of 100 nm was deposited for the device fabrication. Information about the thickness and the atomic bonding of each thin film was confirmed through the field-emission scanning electron microscope (FE-SEM, Hitachi S-4800, Hitachi High-Tech Corporation, Tokyo, Japan) image and X-ray photoelectron spectroscopy (XPS, Nexsa, Thermo Fisher Scientific, Waltham, MA, USA). The Keithley 4200-SCS (Tektronix, Beaverton, OR, USA) semiconductor parameter analyzer was cited for the verification of electrical properties, and the 4225-PMU ultrafast module was connected to the 4200-SCS to implement the synaptic function.

## 4. Conclusions

To figure out the application in both the short term and the long term, two different types of I-V curves, dynamic and static, were confirmed. At room temperature, the retention in the dynamic curve was slowly degraded, while in static, it maintained well for 10^3^ s. To consider the formation and rupture of filaments, the linear fitting of conduction mechanisms was conducted to each curve. It was found that Sn^4+^ had a profound effect on switching, and then the synaptic function was mimicked with a rectangular-shaped pulse. PPF was implemented successfully and showed that the interval between pulses promotes the plasticity of the device. Lastly, the conversion from STP to LTP was achieved by utilizing the spiking signals and showed that the memory property could be applied as the synaptic device. Therefore, we concluded that the device stacks of ITO/SnO_X_/TaN could be working as a good candidate for neuromorphic networks.

## Figures and Tables

**Table 1:** Percentage of oxygen–tin bonds in ITO/SnO_X_/TaN RRAM device. Area was calculated at the core level of O 1s and Sn 3d, respectively.

Etch Time	Area Ratio O-Sn^2+^	O-Sn^4+^	Area Ratio Sn^2+^	Sn^4+^	Ratio of Sn^2+^/Sn^4+^
200 s	46.3%	44.8%	53.9%	32.0%	1.68
248 s	36.0%	50.2%	51.9%	45.6%	1.14

## Data Availability

Not applicable.
